# Representativeness and face-ism: Gender bias in image search

**DOI:** 10.1177/14614448221100699

**Published:** 2022-06-19

**Authors:** Roberto Ulloa, Ana Carolina Richter, Mykola Makhortykh, Aleksandra Urman, Celina Sylwia Kacperski

**Affiliations:** GESIS—Leibniz Institute for the Social Sciences, Germany; University of Passau, Germany; University of Bern, Switzerland; University of Zurich, Switzerland; University of Mannheim, Germany; Seeburg Castle University, Austria

**Keywords:** Algorithm auditing, face-ism, gender bias, image search, search engines

## Abstract

Implicit and explicit gender biases in media representations of individuals have long existed. Women are less likely to be represented in gender-neutral media content (representation bias), and their face-to-body ratio in images is often lower (face-ism bias). In this article, we look at representativeness and face-ism in search engine image results. We systematically queried four search engines (Google, Bing, Baidu, Yandex) from three locations, using two browsers and in two waves, with gender-neutral (person, intelligent person) and gendered (woman, intelligent woman, man, intelligent man) terminology, accessing the top 100 image results. We employed automatic identification for the individual’s gender expression (female/male) and the calculation of the face-to-body ratio of individuals depicted. We find that, as in other forms of media, search engine images perpetuate biases to the detriment of women, confirming the existence of the representation and face-ism biases. In-depth algorithmic debiasing with a specific focus on gender bias is overdue.

Our ability to form impressions about others relies on information that is provided under uncertainty, so we use heuristics to make judgments and decisions (Tversky and Kahneman, 1974). Such impression formation is associated with stereotyping: images, for example, can and often do embody and perpetuate gender stereotypes (Coltrane and Adams, 1997; Len-Ríos et al., 2005; Rodgers and Thorson, 2000). Visual representations of women and men shape our mental representations of gender roles and can reinforce or stabilize them—digital and analog media such as newspapers, magazines, television, and social media can ascribe strict roles in portrayals, and depict sexual objectifications of women to a much larger degree than of men (Courtney and Lockeretz, 1971; Döring et al., 2016; Miller, 1975; Myers and Biocca, 1992; Zotos and Tsichla, 2014). But even simple stylistic disparities in the portrayal of men and women can already subtly perpetuate gender biases (Archer et al., 1983; Blumberg, 2008; Grau and Zotos, 2016). For example, the representation of women politicians in online media influenced how competent and likable they were perceived by voters (Bligh et al., 2012), and variations in facial prominence led to women being rated lower on intelligence (Archer et al., 1983).

The automatic processing of large amounts of potentially biased information has been a topic of particular interest in communication research in recent years (Goldman, 2008; Noble, 2018; O’Neil, 2017). Digital culture and new media studies are moving data to the center of their academic narratives; specifically, data from media platforms (such as social media and search engines) are now considered cultural research objects (Schäfer and Van Es, 2017). In the context of this new paradigm, the question of algorithmic transparency and accountability is often at the forefront of a debate about the utilization of platforms, though often considered unsolvable due to their “black box” dependencies, heterogeneity and embeddedness in wider systems (Crawford, 2016; Kitchin, 2017; Röhle, 2012). Search engine providers, such as Google or Bing, are under academic scrutiny due to their role as gatekeepers: they decide on the relevance of content by filtering and ranking sources of information available on the web (Laidlaw, 2010; Schulz et al., 2005; Wallace, 2018). The output of these decisions shapes our social reality (Just and Latzer, 2017; Noble, 2018) and their implications for citizens and institutions need to be carefully reflected (Aragona, 2021; Halavais, 2018; Thomas et al., 2018). However, the algorithmic choices are not openly accessible (Kitchin, 2017; Paßmann and Boersma, 2017), making independent audits of the outputs crucial.

This study contributes to the research on gender biases in image representations by auditing four search engines’ image search pages. To our knowledge, it is the first time that facial prominence (Archer et al., 1983) is studied in the context of search engines; our work also introduces an approach for automatized estimation of the face-to-body ratio. Finally, we extend on research of representation biases in image search (Araújo et al., 2016; Kay et al., 2015; Magno et al., 2016; Makhortykh et al., 2021; Metaxa et al., 2021; Otterbacher et al., 2017) by comparing multiple search engines on the prevalence of women and men in searched images. We find that image search perpetuates biases to the detriment of women.

## Face-ism and representation biases

The face-ism bias refers to a disparity in facial prominence when representing women and men in visual media, with men typically portrayed with greater facial prominence. The term face-ism, together with the face-ism index (the ratio of the face to the total visible body) was first introduced by Archer et al. (1983). Since then, face-ism has been a topic of study in the context of art and magazines (Dodd et al., 1989), in the online self-representation of politicians (Konrath and Schwarz, 2007; Szillis and Stahlberg, 2007), and in social media, for example, in online profile pictures (Sczesny and Kaufmann, 2018; Smith and Cooley, 2012) and in posted images in social networks (Cifre et al., 2021; Powell, 2016).

Face-ism matters because there is correlational and experimental evidence of a feedback loop between higher facial prominence of a target and higher ratings of agentic traits (intelligent, ambitious, assertive, dominant) as well as a general global positivity effect, including higher likeability (Archer et al., 1983; Levesque and Lowe, 1999; Schwarz and Kurz, 1989; Zuckerman, 1986; Zuckerman and Kieffer, 1994). Typically, women are portrayed with a higher proportion of their body visible, which has been interpreted as a subtle type of sexism in the above studies; crucially, such subtle forms of stereotypes might outlast those that are blatant and receive public attention (Levesque and Lowe, 1999).

In addition to face-ism, gender bias can also be investigated via representativeness, that is, the number of instances of each gender represented in a sample. Gender representation biases have been widely studied in a variety of (new) media, for example, in the news, where topics such as politics and business have been shown to feature women less prominently (Rao and Taboada, 2021). In movies, analyses of frames point to a ratio of 2.5:1 screen occupancy of men compared to women (Jang et al., 2019), with differences in terms of movie genres and a positive evolution toward a more balanced representation in recent years (Mazières et al., 2021). Images from digital platforms Twitter, *The New York Times*, and Wikipedia have also been found to be biased, with women under-represented across a variety of work-related queries such as computer programmer, civil engineer, nurse, and librarian when compared with US labor statistics (Singh et al., 2020).

In this article, we contribute to this literature by including an investigation of representativeness of women and men in search engine image results, as search engines have been shown to reinforce harmful racial and gender stereotypes biases in their algorithms (Noble, 2018), for example, in the existence of negative stereotypes about black women in terms of physical attractiveness (Araújo et al., 2016) and a gendered representation of AI that is biased toward a white anthropomorphization (Makhortykh et al., 2021). Specifically for image search on gender bias, Otterbacher et al. (2017) found that women were over-represented in Bing image searches related to warm traits (e.g. “emotional”), and under-represented in agentic traits (e.g. “rational”). Similarly, researchers have shown stereotype exaggerations in gender representation in Google image searchers for occupations; for example, women were over-represented in images for “nurse” searches, but under-represented for “doctor” searches (Kay et al., 2015), a state which has not improved as evidenced by findings of a similar study five years later (Metaxa et al., 2021).

## Search media and society

In the conceptualization of new media, Manovich (2003) lists eight definitions; a page of search results as a technological object fits them all (Metaxa et al., 2019). Search results are generated by algorithms, which are discussed in the cultural studies and new media literature from a variety of perspectives with the aim to better understand their relevance, implications for ethics and their impact on our lives and institutions (Kitchin, 2017; Napoli, 2014; Paßmann and Boersma, 2017). While algorithms are simply presented as a set of rules, it has been highlighted that these rules are shaped by decisions, politics, and ideologies (Gillespie et al., 2013; Kitchin, 2017), and that algorithms replicate existent biases on the annotated data sets used in their training (Hajian et al., 2016).

While much has been said about the impact of transparency and debiasing (Ananny and Crawford, 2018; Crawford, 2016; Lee, 2018; Paßmann and Boersma, 2017; Shin and Park, 2019; Wijnhoven and Brinkhuis, 2015), and how to shed further light on the “black boxes” of algorithms (Röhle, 2012), some researchers argue that it might be sufficient to develop tools and skills which allow researchers to circumvent the need to understand all processes underlying algorithms (Epstein et al., 2017; Schäfer and Van Es, 2017; Wijnhoven and Brinkhuis, 2015). Furthermore, transparency by itself might be inadequate, with the idea that analyzing the algorithm or the underlying data is insufficient to hold the algorithm accountable, never mind the system behind it (Ananny and Crawford, 2018). Arguments have been made for a stronger oversight over the design and programming process, for which individuals and the institutions or corporate organizations currently hold agency and should therefore hold responsibility (Ames, 2018; Klinger and Svensson, 2018). Power relations on both an individual and structural perspective need to be considered in order to improve accountability across the current technological system (Klinger and Svensson, 2018). Unfortunately, resource allocation seems to be largely carried out to increase relevance in terms of market factors, and quieten the technological concerns, with determinants of quality of the media content at best secondary, at worst irrelevant (Van Couvering, 2007). The assessment of algorithm quality is done by anonymous actors subject to their own cultural norms (Bilić, 2016), and decisions are undertaken in an environment that promotes the obfuscation of details on how relevance is assigned in algorithms to avoid users gaming the system (Gyongyi and Garcia-Molina, 2005; Malaga, 2008). In sum, search engine companies adjust and evaluate their algorithms with their own sets of goals and values, attending to diverse cultural contexts, and utilizing a variety of person groups: as a result, different search engines produce very different outputs even when all other factors are held constant (Makhortykh et al., 2021).

As an additional line of inquiry, the debate regarding the importance of algorithm fairness, transparency and accountability (Kemper and Kolkman, 2019; Kroll et al., 2017; Lee, 2018; Lepri et al., 2018; Wieringa, 2020) has inspired research on how digitalization has intensified mediatization, that is, the embedding of media in social processes (Andersen, 2018; Couldry and Hepp, 2018). In terms of search engines, for example, selection and rankings are used by individuals as a measure for content quality and relevance (Edelman, 2021; Keane et al., 2008; Schultheiß et al., 2018; Urman and Makhortykh, 2021), influencing judgments, decisions and behavior related to aspects as diverse as correctness of health information (Kammerer and Gerjets, 2012; Lau and Coiera, 2009), voting preferences (Epstein et al., 2017; Epstein and Robertson, 2015; Zweig, 2017), purchases (Ghose et al., 2014), and writing (Novin and Meyers, 2017).

Following these concerns and influence mechanisms, researchers are systematically reviewing search engine outputs through algorithmic auditing (Bandy, 2021; Mittelstadt, 2016). Not surprisingly, prominent companies and their platforms have drawn the most attention: in a recent literature review, almost half of the reviewed papers focused on Google (Bandy, 2021). In the midst of controversies (Metz, 2021; Wakabayashi, 2017), and scrutiny (Bilić, 2016; Noble, 2018), Google is seemingly pushing to improve their image as manifested by their work on increasing search transparency (Beutel et al., 2019; Bird et al., 2019; Mitchell et al., 2020; Schumann et al., 2021) and diversity (Google, 2021). The academic attention drawn to Google seems justified because of its dominance while/whereas the search engine market (~92%), where Bing occupies second place (~2.5%); however, other search engines can serve as comparison points and are important actors in their respective markets: Baidu as the dominant search engine for the Chinese market (~76%), and Yandex, which holds ~43% of the market share in Russia (Statcounter, 2021).

Noble (2018) has described the societal impact of gender and racially biased algorithms with an emphasis on the outputs of search engines and recommendation systems. Image search is of particular relevance because images are more memorable (Nelson et al., 1976), are often searched for to explore or learn (Xie et al., 2018), and have a stronger potential in shaping public opinion (Bleiker, 2018; Grabe and Bucy, 2009; Powell et al., 2015). In line with previous evidence, Kay (2015) demonstrated that image search results can shift individual’s perception about gender distributions in occupations by ~7%, with further data supporting the idea that individuals with sexist attitudes are less likely to recognize gender-biased results (Otterbacher et al., 2018). This article is positioned at the intersection of the above lines of research, attempting to provide a further argument as to how search engine providers can increase their accountability for biased image results, with a specific focus on search engine algorithms as vehicles that shape social reality and provide utility for users (Halavais, 2018; Thomas et al., 2018).

## Biases in image recognition

Advances in machine learning allow for the recognition of the elements (e.g. faces, individuals) and attributes (e.g. gender, age group of individuals) that compose the image. Given the size, constant evolution and computational requirements of such machine learning models, companies have started providing access to these models via APIs instead of providing software packages that can be run on one computer.

Researchers have demonstrated the presence of biases in these APIs, which can lead to asymmetric performance depending on gender and race (Benjamin, 2019; Buolamwini and Gebru, 2018). Performance errors are often attributed to biases in the datasets used to train the models (Hajian et al., 2016), though balanced datasets have been shown to be insufficient on their own, with mechanisms within the algorithm possibly also leading to biases (Wang et al., 2019). The process of image annotation is rarely described, and race and gender as attributes are often simplified and portrayed to be insignificant, indisputable, and apolitical (Scheuerman et al., 2020). Because of this simple conceptualization of attributes, image recognition systems provide a binary classification for gender (Hamidi et al., 2018; Keyes, 2018) and have been shown to be erroneous on transgender individuals (Scheuerman et al., 2019). The exclusion of particular groups in these algorithmic systems might have broader implications for the individuals mental’s health when applications are built on top of these technologies, such as the case of inferred gender markers used in image filtering (Goetz, 2021).

Given that our methodology leverages the Amazon Web Services (AWS) Rekognition API to automatize the annotation, we make sure to evaluate its performance. In addition, in the discussion, we extend on the implications of the use of a nonbinary classification of gender for auditing processes.

## Present research and hypotheses

In this study, we advance the existing scholarship on biases in image search results as outlined in the previous sections by investigating gender biases, using a systematic auditing approach to control for multiple forms of noise. Specifically, we extend previous findings of the representation bias in image search (Araújo et al., 2016; Kay et al., 2015; Magno et al., 2016; Makhortykh et al., 2021; Metaxa et al., 2021; Otterbacher et al., 2017) to four major search engines (Google, Bing, Baidu, and Yandex). For the first time, we investigate whether the face-ism bias (Archer et al., 1983) can be observed in search engine image results. Methodologically, we introduce a novel approach for automatized estimation of the face-to-body ratio.

To do so, we conducted two experiments. The first builds on research by Otterbacher et al. (2017) and investigates the image results for the nongendered query “person,” as well as, to capture an agentic trait, “intelligent person.” In a second experiment, gendered queries “woman” and “man” as well as “intelligent woman” and “intelligent man” are employed. We also explored the moderating effect of search engines, expecting differences between Google, Bing, Baidu, and Yandex due to evidence that their outputs differ even when all other factors are held constant (Makhortykh et al., 2021).

In the first experiment, we hypothesized that images with male faces would be over-represented, and that the size of the effect would differ depending on the search engine (H1). We expected the inclusion of “intelligent” to increase this overrepresentation further (H2). Moreover, we hypothesized that searching for “person” would result in a larger face-to-body ratio for images with male faces as compared with images with female faces (i.e. the face-ism effect); and that this would again differ between search engines (H3). Once more, we expected the added adjective “intelligent” to strengthen this effect (H4).

In our second experiment, for the gendered queries “man” and “woman,” we hypothesized a higher face-to-body ratio for images with male faces as compared with images with female faces (i.e. the face-ism effect), and that this would differ between search engines (H5). We here also expected that the inclusion of the adjective “intelligent” would strengthen the face-ism bias (H6).

## Methodology

### Image collection

There are several considerations when it comes to auditing search engine results: (1) personalization, that is, the adjustment of search results according to user characteristics (Hannak et al., 2013) such as the location from which the searches are requested (Kliman-Silver et al., 2015), the previous browsing history (Haim et al., 2018; Mikians et al., 2012; Robertson et al., 2018) or the user profiles which can include the individual’s gender (Wijnhoven and Van Haren, 2021); (2) randomization, that is, unexplained differences that emerge even under the seemingly equal browsing conditions (location, browser type, incognito mode); and (3) time effects, that is, the adaptation of results according to the historical context at play during the data collection (Metaxa et al., 2019; Urman and Makhortykh, 2021).

Our methodology (Ulloa et al., 2021) controls for these factors by simulating user behavior of 240 agents (one agent corresponds to one automatized browser) with different IP addresses, on two Internet browsers types (Firefox and Chrome), sending the same query term synchronously from different locations (see below), removing historical browser data (e.g. cache, cookies) before performing each query, and by repeating the data collection in two different waves (on March 12 and 17, 2021). Ten agents under the same condition (combination of browser, region and search engine) entered queries into the assigned search engine and saved the resulting html. The experience of each agent can be likened to that of users when navigating via a “private” browser mode. We then extracted image URLs and retrieved the corresponding images.

We chose Google, Bing, Baidu and Yandex due to their large market shares in their respective regions (Statcounter, 2021). The server locations (London, UK; Ohio, US; North California, US) were selected based on the availability of AWS servers (from which the infrastructure is launched). We utilized countries with the same language (English) to avoid confound between language and regions, and to match it to the language of the query terms. Although we included search engines Baidu and Yandex that hold major shares in the Chinese and Russian markets, the experiments were constrained to one language only, due to infrastructure costs. We selected English for its relevance in comparing our findings with prior research (such as Otterbacher et al., 2017).

The agents scrolled search result pages until at least the first 100 images^1^ for each query appeared in the browser. We used six queries: “person,” “intelligent person,” “woman,” “man,” “intelligent woman,” “intelligent man.” The procedure was repeated ten times for each of the queries to account for search randomization; duplicates within these 10 repeats were eliminated.

Of the 30,043 images retrieved, those without a face (17.14%) and those with multiple faces (14.08%) were excluded from analysis. Number of images with at least one face per query and search engine can be seen in Table 1.

**Table 1.** Total images with one face based on the top-100/agent. The first column presents search engines. Further columns list query terms. Each cell contains the total number of images analyzed per engine and query (for all waves, locations, and browsers). Duplicates of images within the same condition were removed.

	Person	Woman	Man	Intelligent person	Intelligent man	Intelligent woman
Google	802	1054	1114	562	786	682
Bing	1290	1353	1444	1030	1063	1068
Baidu	282	879	628	196	230	360
Yandex	1051	1288	1062	832	987	1105

### Design and data analysis

Collected images were analyzed via the Amazon Rekognition API provided by AWS, which annotates images for a variety of content features, including the presence/absence of persons, presence/absence of faces, area of persons’ faces, and area of persons’ bodies. It also provides tags with gender information based on visual clues (i.e. gender expression, coded in a binary manner as female/male).

To test the reliability of feature detection, a trained research assistant checked all images to detect false positives and false negatives. The performance metrics are reported in Table 2. Although precision was high, we manually corrected false positives.

**Table 2.** Performance metrics of the Amazon Rekognition service. The first column presents a tag identifier and number (*N*) of unique images that were tagged. Following this are performance metrics used in pattern recognition: true positives (TP), true negative (TN), false positives (FP), false negatives (FN), precision (proportion of correctly tagged images), recall (proportion of cases associated to the tag that were correctly retrieved), and *F*-score (the harmonic mean of precision and recall). We also indicate the metric values obtained after correcting the FP in brackets. The face-ism row evaluates the cases in which the face and person was correctly detected and associated (i.e. the face tag being inside the body tag).

Tag (*N*)	TP	TN	FP	FN	Precision	Recall	*F*-score
Face (4039)	3229	794 (802)	8 (0)	56	.998 (1)	.983	.990 (.991)
Face-ism (3237)	3137	44 (49)	5 (0)	51	.998 (1)	.984	.991 (.992)
Female face (3237)	1483	1577 (1702)	125 (0)	52	.922 (1)	.966	.944 (.983)
Male face (3237)	1630	1427 (1535)	108 (0)	72	.938 (1)	.958	.948 (.978)

Presence of a face was coded as 1 (if present) or 0 (if not present), that is, dummy coded. The face-ism index was calculated as the ratio of head height divided by body height (continuous value between 0 and 1) (Archer et al., 1983). The height of the face and the entire person were supplied via coordinates by AWS. Face-ism was only calculated for images in which face and body were annotated as belonging to the same individual (i.e. AWS detected a face area contained inside the body area with 5% tolerance based on the body dimensions; face-ism tag in Table 2).

For our first experiment, we followed a 2 (gender) × 2 (person vs intelligent person) × 4 (Google, Bing, Yandex, Baidu) design. The dependent variables were representation bias (presence/absence of a face) and the face-to-body ratio (higher value corresponds to less face-ism). For our second experiment, we followed a 2 (gender) × 2 (man/woman vs intelligent man/woman) × 4 (Google, Bing, Yandex, Baidu) design to examine face-to-body ratio.

All analyses were conducted controlling for browser (Chrome vs Firefox), location (US1 vs US2 vs UK), and data collection wave (waves 1 vs 2). We found no significant effects of the three control factors across any of our analyses, so they will not be further discussed in the following sections.

Statistical analyses were conducted using the programming language for statistical computing R. We used (1) linear regressions (lm package) to model the relationship between our response variables (face presence, and face-ism index) and factors (engine, gender, query term); (2) analysis of variance (ANOVA, aov package) to report overall statistical differences in the models; and (3) contrasts (emmeans package) for multiple comparisons of specific treatments (i.e. combinations of engine, gender, and query term). For the models, we report *p*-value (contrast is considered statistically significant if it is below a threshold of 0.05), χ^2^ and odds ratios for binomial linear regressions (i.e. representation), and *F* statistics for Gaussian linear regressions (i.e. face-ism index). In the contrast tables, we also report the standard error, *z*-ratio (measurement of the contrast in relationship to the global mean along the base of a normal distribution, also called *z*-distribution), 95% confidence intervals and Cohen’s *D* (to estimate the effect size).

## Results

### Experiment 1: nongendered search

We used a binomial linear model to predict image representativeness (coded 0,1) from an interaction of gender (female, male), query (person, intelligent person), and search engine. We found a significant triple interaction, χ^2^ (3, *N* = 6045) = 160.230, *p* < .001. Table 3 shows relevant post hoc contrasts for gender, query, and search engine.

**Table 3.** Post hoc contrasts for number of faces in the person queries. Effects of gender by query and search engine and of the gender × query interaction by search engine on image representativeness. *Results averaged over the levels of region, browser, wave. Main effects are adjusted for interaction influence and multiple comparison (Tukey). Gender coded: 0* *=* *women; 1* *=* *men. Bolded rows were found significant at the .05 level. Negative z-ratio indicates lower representation of women. For example, at odds ratio (OR) 0.23, Bing was four times as likely to show images of men than of women in the person query, while at OR of 4.5, Baidu was 4.5 times more likely to show images of women than of men in the person query.*

	Search engine	Odds ratio	Standard error	*z*-ratio	*p*-value	95% CI L	95% CI H
Gender—Person	**Baidu**	**4.551**	**0.822**	**8.388**	**<.001**	**2.861**	**7.239**
	**Bing**	**0.228**	**0.019**	**−17.553**	**<.001**	**0.184**	**0.283**
	Google	1.233	0.123	2.096	.154	0.954	1.595
	**Yandex**	**0.043**	**0.005**	**−27.178**	**<.001**	**0.032**	**0.058**
Gender—Intelligent person	Baidu	0.721	0.146	−1.614	.37	0.428	1.213
	**Bing**	**0.146**	**0.014**	**−19.511**	**<.001**	**0.114**	**0.188**
	**Google**	**0.188**	**0.024**	**−12.866**	**<.001**	**0.135**	**0.263**
	**Yandex**	**0.098**	**0.011**	**−20.216**	**<.001**	**0.073**	**0.131**
Gender × Query	**Baidu**	**0.158**	**0.043**	**−6.786**	**<.001**	**0.093**	**0.27**
	**Bing**	**0.641**	**0.083**	**−3.427**	**.001**	**0.498**	**0.827**
	**Google**	**0.152**	**0.025**	**−11.473**	**<.001**	**0.111**	**0.21**
	**Yandex**	**2.253**	**0.368**	**4.981**	**<.001**	**1.637**	**3.102**

**Figure 1.**
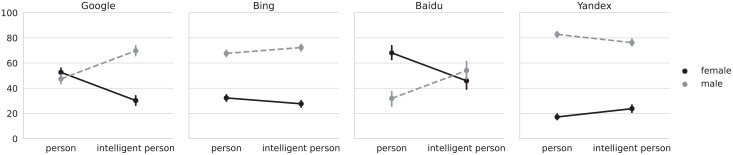
Percentage of images for either gender (female/male), for the “person” and “intelligent person” queries, by search engine. The four plots correspond to each of the analyzed search engines. The *Y*-axis shows the percentage of pictures obtained by the query terms (“person” and “intelligent person”) used to retrieve the results from the search engine that are indicated in the *X*-axis. The legend indicates the color, to distinguish between the pictures corresponding to female faces (black continuous line) or male faces (gray dashed line). Vertical lines crossing the data points indicate bootstrapped 95% confidence intervals. For example, in the left plot (Google), ~50% of pictures show female faces for the query term “person,” and ~50% show male faces. In the same plot, ~30% of pictures show female faces for the query term “intelligent person,” while ~70% of pictures show male faces.

Post hoc contrasts revealed that the odds of seeing an image with a female face (as opposed to male face) when searching for “person” were significantly lower for Bing and Yandex. For Baidu, we found the opposite to be true, that is, images with female faces were significantly more common. We did not find a significant difference for Google. This changed when searching for “intelligent person”: the interaction of query and gender was found to be significant across all four search engines. As can be seen in Figure 1, adding the adjective “intelligent” increased the odds of seeing images with male faces for Google, Bing, and Baidu, while decreasing the odds of seeing images with female faces, previously called a backlash effect (Otterbacher et al., 2017); for Yandex, the interaction was reversed. H1 and H2 were therefore partially supported.

We then predicted the face-to-body ratio from the interaction of gender (female, male), the query (“person” vs “intelligent person”) and search engine with a linear model; we found a small significant triple interaction, *F*(3, 5924) = 23.03, *p* < .001. Table 4 holds results from relevant post hoc contrasts for gender, query, and search engine.

**Table 4.** Post hoc contrasts for face-ism for the person queries. Effects of gender by query and search engine and of gender × query interaction by search engine on face-ism. *Results averaged over the levels of region, browser, wave. Main effects are adjusted for interaction influence and multiple comparison (Tukey). Gender coded: 0* *=* *women; 1* *=* *men. Effect size is Cohen’s D. Bolded rows were found significant at the .05 level. Negative z-ratio indicates lower representation of women.*

	Search engine	Estimate	Standard error	*z*-ratio	*p*-value	95% CI L	95% CI H	Effect size
Gender—Person	**Baidu**	**−0.173**	**0.026**	**−6.616**	**<.001**	**−0.24**	**−0.106**	**0.888**
	Bing	−0.027	0.012	−2.336	.09	−0.057	0.003	0.140
	**Google**	**−0.064**	**0.014**	**−4.637**	**<.001**	**−0.099**	**−0.028**	**0.328**
	**Yandex**	**0.08**	**0.016**	**5.019**	**<.001**	**0.039**	**0.121**	**0.410**
Gender—Intelligent person	Baidu	0.053	0.028	1.912	.223	−0.018	0.125	0.274
	**Bing**	**−0.043**	**0.014**	**−3.17**	**.008**	**−0.078**	**−0.008**	**0.221**
	**Google**	**−0.202**	**0.019**	**−10.626**	**<.001**	**−0.251**	**−0.153**	**1.040**
	Yandex	0.014	0.016	0.902	.804	−0.026	0.055	0.074
Gender × Query	**Baidu**	**0.226**	**0.038**	**5.918**	**<.001**	**0.151**	**0.301**	**1.162**
	Bing	−0.016	0.018	−0.889	.374	−0.051	0.019	0.082
	**Google**	**−0.138**	**0.023**	**−5.894**	**<.001**	**−0.184**	**−0.092**	**0.712**
	**Yandex**	**−0.065**	**0.022**	**−2.918**	**.004**	**−0.109**	**−0.021**	**0.337**

**Figure 2.**
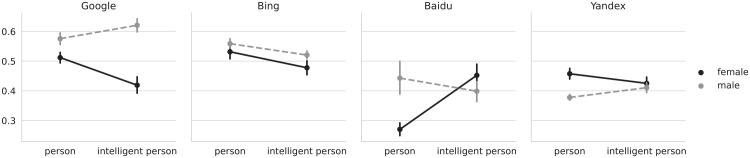
Face-ism of images for either gender (female/male) or for the “person” and “intelligent person” queries, by search engine. The four plots correspond to each of the analyzed search engines. The *Y*-axis shows the average face-ism index of pictures obtained by the query terms (“person” and “intelligent person”) used to retrieve the results from the search engine that are indicated in the *X*-axis. The legend indicates the color, to distinguish between the pictures corresponding to female faces (black continuous line) or male faces (gray dashed line). Vertical lines crossing the data points indicate bootstrapped 95% confidence intervals. *Y*-axis is truncated to fit the minimum and maximum values. As an example, in the left plot (Google), the face-ism index was >.5 for the query term “person” when the image showed a female face, whereas it was <.6 when the image showed a male face; for the query term “intelligent person” the face-ism index was >.4 for images that showed a female face, while it was >.6 for images that showed a male face. A lower face-ism index means that the image showed more of a person’s body.

The contrasts reveal that, averaged over all other levels, Google and Baidu show a lower face-to-body ratio for women for the “person” query. For Yandex, men have the lower face-to-body ratio. We did not find a significant difference for Bing. We also found the interaction of gender and query to be significant for Baidu, Google and Yandex. As can be seen in Figure 2, for Google and Yandex, adding the term intelligent to the query increased face-ism; for Baidu, the interaction was reversed. We found no significant interaction for Bing. H3 and H4 were therefore partially supported.

### Experiment 2: gendered search

For this experiment, we used queries “woman,” “intelligent woman,” “man,” and “intelligent man.” We predicted the face-to-body ratio from an interaction of gender (female, male), the query (adjective: none, intelligent) and search engine with a linear model; we found a small significant triple interaction *F*(3, 14366) = 9.07, *p* < .001. Table 5 holds results from relevant post hoc contrasts for gender, query, and search engine.

**Table 5.** Post hoc contrasts for face-ism for the gendered queries. Effects of gender by query and search engine and of gender × query interaction by search engine on face-ism. *Results averaged over the levels of region, browser, wave. Main effects are adjusted for interaction influence and multiple comparison (Tukey). Gender coded: 0* *=* *women; 1* *=* *men. Effect size is Cohen’s D. Bolded rows were found significant at the .05 level. Negative z-ratio indicates lower representation of women.*

	Search engine	Estimate	Standard error	*z*-ratio	*p*-value	95% CI L	95% CI H	Effect size
Gender—Woman/Man	Baidu	−0.028	0.011	−2.472	.064	−0.057	0.001	0.149
	**Bing**	**−0.077**	**0.007**	**−10.621**	**<.001**	**−0.095**	**−0.058**	**0.404**
	**Google**	**−0.026**	**0.008**	**−3.161**	**.009**	**−0.047**	**−0.005**	**0.137**
	**Yandex**	**0.049**	**0.008**	**6.240**	**<.001**	**0.029**	**0.070**	**0.260**
Gender—Intelligent woman/man	Baidu	0.042	0.018	2.380	.081	−0.003	0.087	0.221
	**Bing**	**−0.107**	**0.009**	**−12.457**	**<.001**	**−0.129**	**−0.085**	**0.563**
	Google	0.003	0.01	0.298	.991	−0.023	0.029	0.016
	**Yandex**	**0.026**	**0.008**	**3.080**	**.011**	**0.004**	**0.047**	**0.136**
Gender × Query	**Baidu**	**0.070**	**0.021**	**3.340**	**.001**	**0.029**	**0.111**	**0.369**
	**Bing**	**−0.030**	**0.011**	**−2.682**	**.007**	**−0.052**	**−0.008**	**0.258**
	**Google**	**0.029**	**0.013**	**2.230**	**.026**	**0.004**	**0.054**	**0.153**
	**Yandex**	**−0.024**	**0.012**	**−2.048**	**.041**	**−0.046**	**−0.001**	**0.124**

**Figure 3.**
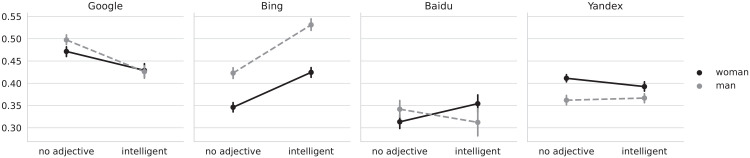
Face-ism of images for the “woman/man” and “intelligent woman/man” queries, by search engine. The four plots correspond to each of the analyzed search engines. The *Y*-axis shows the average face-ism index of pictures obtained by the query terms used to retrieve the results from the search engine. The query terms are represented in the *X*-axis (adjective) and the legend (noun), for example, “no adjective” in the *X*-axis and “woman” on the legend correspond to the “woman” query term, whereas “intelligent” and “woman” to the “intelligent woman” query term. The legend indicates the color, to distinguish between the queries corresponding to woman- and man-related queries. Vertical lines crossing the data points indicate bootstrapped 95% confidence intervals. *Y*-axis is truncated to fit the minimum and maximum values.

For the base queries only, the contrasts for Google and Bing show, averaged over all other levels, a lower face-to-body ratio for images of women. For Yandex, we found images of men to have the lower face-body ratio. We did not find a significant difference for Baidu. We also found the interaction of gender and query to be significant across all four search engines. As can be seen in Figure 3, for Google, the face-to-body ratio decreased slightly stronger for men than for women. For Bing, the ratio increased for both men and women; for Baidu, the face-to-body ratio increased for women, while decreasing for men; and for Yandex, it decreased slightly for women. H5 and H6 were therefore partially supported.

## Discussion

Gender bias was investigated in images of women and men across four search engines: Google, Bing, Baidu, and Yandex. First, we looked at representation bias, that is, the number of images showing women and men when searching for gender-neutral terminology (“person”); second, we looked at the face-ism bias, that is, lower face-to-body ratio in images of women compared with those of men, in both gender-neutral and gendered queries.

Overall, our main hypothesis that image search outputs tend to be biased, most frequently to the detriment of women, was confirmed. Some variability exists for certain queries and search engines, but a pattern can be observed: across the 24 main effect contrasts (see Tables 3–5), 13 were significantly biased against women. Seven did not show significant differences, and in four cases, images showing men were more biased.

In terms of specific findings, it is first important to note the lower number of images showing women compared with men when searching for “person” and “intelligent person.” This aligns with previous evidence for Bing with a variety of agentic traits (Otterbacher et al., 2017), across different professions (Kay et al., 2015). Our data show that Bing and Yandex are particularly biased for gender-neutral queries and a backlash toward women is observed when adding an agentic trait (“intelligent person”) for both Google and Baidu.

Second, we observed the face-ism bias in search engines that dominate the global market (Google, Bing) for the “person” and “woman/man” queries. No clear pattern emerged when adding the qualifier intelligent. For Google, a backlash effect was visible for the “person” search; Baidu images also showed face-ism for both “person” and “woman/man” queries, but differences disappeared when adding “intelligent.” For Yandex, we found the opposite, that is, a lower face-body ratio for men, but a backlash toward women when adding the qualifier. These differences suggest that generalizations about the presence or absence of bias in search engine outputs should be made with caution and future research should judge them on a case-by-case basis.

Two results are deserving of further discussion. On one hand, for Google as the search engine with the largest market share, we found that for the base query “person,” the odds of seeing an image of a woman or a man were even—the most unbiased of the four search engines. This makes sense as previous works that call for bias reduction have mainly focused on Google (Bandy, 2021; Kay et al., 2015; Noble, 2018). While this effort is commendable, our analysis shows that in this case, output debiasing remains superficial, and biases resurface upon further scrutiny, that is, when adding “intelligent” to the query.

On the other hand, for Yandex, we found that the face-to-body ratio was reversed, with images of men showing a lower ratio. It is unclear whether this is due to algorithmic choices made by Yandex or is based in cultural differences in the representation of women’s and men’s faces in Eastern Europe. Especially in light of previous evidence indicating that more gender equal societies might be prone to more face-ism bias (Konrath et al., 2012), the result prompts the need for further investigation, by, for example, including other languages and locations in the analysis.

This article also contributes methodologically: to our knowledge, ours is the first investigation in which face-ism is estimated automatically by trained machine learning models, via the AWS Image Rekognition API. We demonstrated that the model performs well at this task—we did not observe a change in results before and after human correction. The automatization greatly simplifies operationalization of face-ism detection. This is important in the context of previous attempts to address biases, for example, attempts to alert users to biased search results (Epstein et al., 2017), the provision of dialectic search that presents alternative views (Wijnhoven and Brinkhuis, 2015; Wijnhoven and Van Haren, 2021), or the design of better training datasets for the underlying algorithms (Buolamwini and Gebru, 2018). Our approach thus also yields an opportunity for debiasing, for example, by including the face-ism index in image selection processes.

Overall, our findings contribute to the research on accountability and utility of algorithms (Kitchin, 2017; Napoli, 2014; Schäfer and Van Es, 2017). Critically, independent audits have been argued to improve both (Bandy, 2021; Kitchin, 2017; Raji et al., 2020), as providing evidence of biases in search engine image results can serve as a stepping stone for search engine providers to provide higher-quality results and a fairer representations in their outputs (Bilić, 2016; Raji and Buolamwini, 2019; Seufert, 2014; Van Couvering, 2007).

In one positive instance, in the wake of a seminal work on racial bias in face recognition technology (Buolamwini and Gebru, 2018), a follow-up impact analysis of the publication of this work revealed that accuracy improved for the three target companies, while it did not for two nontargeted companies (Raji and Buolamwini, 2019). In other instances, the algorithms or tags in question were simply removed following audits (*BBC News*, 2020; Simonite, 2018). Still, the question of the impact evaluation of algorithmic audits remains pertinent (Raji et al., 2020). Advances in regulation, for example, the recent Digital Service Act (European Commission, 2022), will aid this by including obligations with regards to algorithmic transparency: publishing recommender systems’ inner workings, and providing key data to researchers to analyze how online risks evolve, will provide the opportunity to more closely look at those parts of the search value chain (e.g. crawling, indexing, ranking) that are most biased.

### Future directions

It is important to make the results of such audits available to the broader public to increase social awareness about risks inherent in the use of algorithms by citizens, by policymakers, and the institutions they belong to (Aragona, 2021). The challenge here is twofold. First, the impact of biased search engine results, such as representation bias and face-ism that we discuss here, needs to be further investigated. While first research has been carried out on how search engine designs and biases affect individuals (e.g. Epstein and Robertson, 2015; Kammerer and Gerjets, 2012; Kay et al., 2015; Lau and Coiera, 2009; Metaxa et al., 2021), a more structured approach to study the effects of biased image representations on the viewers that consume them can help better understand how to leverage audit results to have a wider impact on awareness in our communities. This is the second challenge: communication of audit results to citizens, who often attribute efficiency and objectivity to algorithmic output (Lee, 2018) and can become overwhelmed with the layers of information that arise as transparency increases (Diakopoulos and Koliska, 2017; Thomas et al., 2018) and with the need to manage awareness of biases and trust (Eslami et al., 2017; Rossi, 2018).

In terms of future academic research based on our contributions, we have three suggestions. First, researchers can explore representation and facial prominence biases in data using search queries related to race, socioeconomic, or even immigrant status (see e.g. Urman et al., 2022), and the intersections between them. Second, the automatic annotation capabilities can be explored beyond the face-to-body ratio reported here, for example, to investigate whether gender biases exist also with regards to area coverage, body positioning, or presence of objects in the image and their relation to the person. Finally, it is worthwhile to study the psychological and behavioral implications of the existence of biases in search engines by studying human attitudes and decision making when exposed to representation and facial prominence biases in an online context.

### Limitations

The reliance on image recognition algorithm in our methodology comes with two limitations. First, the algorithms can be biased (Buolamwini and Gebru, 2018; Raji et al., 2020). To mitigate this, a research assistant inspected the classification for errors, to evaluate the performance of the automatic annotation. We aimed at an annotation strategy that used visual gender expression, however, the research assistants and the authors who supervised the assistant’s work, are also not immune to biases that affect judgment. The exceptionally high inter-rater reliability between the human and machine performance could be taken as an indicator of improvements in the image recognition algorithm, but we are cautious on establishing further conclusions in this direction as this was neither the purpose of this study, nor was the image sample adequate to answer this question. While we demonstrated that women were under-represented, other attributes, such as race and age, might be a confounding factor, as image recognition shows poorer performance in those as well (Buolamwini and Gebru, 2018; Raji et al., 2020).

As a second limitation, we acknowledge the exclusion of nonbinary individuals in the current study. Search engines rely on automatic annotation for their image filtering and display criteria, so it can be expected that algorithms filter out instances that are ambiguous with regards to queries—in other words, images that the algorithm does not tag with “woman” or “man” would not be displayed in search results in the first place. And, with regards to our own use of image annotation to perform analyses, while we would have preferred a continuous gender annotation, the image recognition services we considered all used a binary classification for gender (our decision for AWS was finally taken to avoid Alphabet or Microsoft services, as the latter companies own Google and Bing, respectively).

Research on how gender expressions on a continuous scale are portrayed in search engine image results could enrich the literature of the representation of nonbinary individuals on the Internet; however, while a sizable number of individuals identify or visually express themselves as nonbinary or gender-fluid, and inclusivity across the academic literature is increasing (Brierley, 2000; Matsuno and Budge, 2017; Monro, 2019), the decision to release algorithms capable of annotations that identify marginalized groups needs to be reflected. Critical evaluation as to the consequences of such technologies has already been undertaken with regards to sexual orientation; they are often a threat to individuals’ privacy and safety (e.g. Raji et al., 2020; Wang and Kosinski, 2018). Consequently, Alphabet has, for example, removed gender annotation from their Cloud’s Vision API (Ghosh, 2020).

The contribution of the here presented paper remains relevant. The results confirm the existence of biases within a setting in which a gender dichotomy is a norm, and based on our findings, it might be more accurate to say that representations of gender expressions that incline toward femininity (i.e. are algorithmically tagged as “female”) are penalized by search engines.

Some further limitations should inform the interpretations of results. Image exclusions for Baidu (due to absence of faces, or too many faces) were relatively high, and particularly pronounced for the “person” and “intelligent person” queries, maybe due to Baidu-specific difficulties with retrieving results in English. Because results are reported averaged across browser, location, and waves (for which no significant differences were found), ensuing sample sizes were sufficient for analyses, but results for Baidu should be interpreted with caution. Future studies could look at outputs for queries in the language for the region where the search engine holds a major market share (e.g. Chinese for Baidu or Russian for Yandex). Furthermore, we conducted image searches across only two waves—while we did not find significant differences between the waves, they were only 1 week apart. Collecting data over a longer period will give better insights into time-based output fluctuations. Finally, for the “woman/man” queries, face-ism effect sizes were relatively small. In the future, the validity of findings could be improved with a rank-based approach (e.g. Kulshrestha et al., 2017); this was not a viable option for the current study as image search outputs are presented in a grid that varies according to screen sizes and involves less-structured visual processing (see eye-tracking experiments, Lu and Jia, 2014).

## Conclusion

Image search results generated by the world’s largest search engines remain subject to gender bias: 5 years ago, Otterbacher et al. (2017) showed that for the query “person,” Bing defaulted to images of men twice as often as images of women. This has not changed. And although the results for this query are balanced for Google, the gender parity is nullified by adding the qualifier “intelligent.” Search engines do not fare much better when it comes to facial prominence, with men benefiting more often from higher face-to-body ratios. Some search engine differences surface when we compare global market leaders (Google and Bing) against the local market competitors (Yandex and Baidu), indicating that generalizations across search engines do not necessarily hold. But overall, representation and face-ism biases remain a commonplace occurrence that disproportionately affects women.

## Notes

1. The relatively low cutoff was chosen based on research on online search behavior. While more data might seem desirable at first glance, retrieving a larger number of images could reduce the validity of the results: most users do not browse past the third page of search results (André et al., 2009; Park et al., 2015), examination duration falls by almost 70% as users approach the ninth row or the 50th image (Xie et al., 2017) and click-through/hover actions are close to zero past the 30-image mark (Park et al., 2015).
